# Synthesis, Structure and Antibacterial Activity of Two Novel Coordination Polymers Based on *N*,*N*′-bis(4-carbozvlbenzvl)-4-aminotoluene and Heterocyclic Ligand against *S. aureus*

**DOI:** 10.3390/molecules29091990

**Published:** 2024-04-26

**Authors:** Ziyun Wang, Lun Zhao, Hongwei Jing, Guanying Song, Jiayu Li

**Affiliations:** College of Chemistry, Changchun Normal University, Changchun 130032, China; qx202200053@stu.ccsfu.edu.cn (Z.W.); qx202100047@stu.ccsfu.edu.cn (H.J.); qx202100049@stu.ccsfu.edu.cn (G.S.); lijiayu@stu.ccsfu.edu.cn (J.L.)

**Keywords:** coordination polymer, *N*,*N*′-bis(4-carbozvlbenzvl)-4-aminotoluene, heterocyclic ligand, antibacterial activity

## Abstract

As the use of antibiotics increases, the increasing resistance of bacteria is the main reason for the reduced efficiency of antibacterial drugs, making the research of new antibacterial materials become new hot spot. In this article, two novel coordination polymers (CPs), namely, [Cd_2_(L)_2_(bibp)_2_]_n_ (1) and [Ni(L)(bib)]_n_ (2), where H_2_L = *N*,*N*′-bis(4-carbozvlbenzvl)-4-aminotoluene, bibp = 4,4′-bis(imidazol-1-yl)biphenyl, and bib = 1,3-bis(1-imidazoly)benzene, have been synthesized under solvothermal and hydrothermal condition. Structural clarification was performed through infrared spectrum and single-crystal X-ray diffraction analysis, while thermal analysis and XRD technology were used for the performance assessment of compounds **1** and **2**. In addition, antibacterial performance experiments showed that compounds **1** and **2** have certain selectivity in their antibacterial properties and have good antibacterial properties against *S. aureus*. As the concentration of the compound increases, the inhibitory effect gradually strengthens, and when the concentration of the compound reaches 500 μg/mL and 400 μg/mL, the concentration of the *S. aureus* solution no longer increases and has been completely inhibited.

## 1. Introduction

Infections caused by microorganisms pose a serious threat to human health, and pathogen resistance to antibiotics has been escalating in recent years, resulting in serious health complications [1,2,3]. Scientific advances in nanotechnology and materials science have made it possible to produce new substances which have inherent antimicrobial activity, encouraging deeper applications in new technological fields and meeting the challenges of a more modern society [4,5,6]. In the last decade, metal–organic skeleton materials have received increasing attention for biomedical applications on account of their high porosity, distinctive biodegradability and outstanding biocompatibility [7,8,9,10]. In particular, by means of properly tuning organic linkers or metal clusters, MOFs possess the capability to optimize antimicrobial properties at the molecular level, which gives MOFs a competitive advantage over conventional antimicrobial agents [11,12]. Cobalt(II)-, zinc(II)-, copper(II)- and silver(I)-based MOFs have been synthesized and show some antimicrobial effects. MOFs and composites containing MOFs have broad prospects in the field of antimicrobial materials [13,14,15].

Hu et al. developed a functional 2D MOF nano-enzyme electrochemical sensor for sensitive detection of pathogenic *Staphylococcus aureus* (*S. aureus*) [16]. Under optimal conditions, the present bioassay provided an extensive detection range of 10–7.5 × 10^7^ colony-forming units/mL with a detection limit of 6 CFU/mL, which was superior to most anterior reports [17,18]. A 3D Cu(II) metal–organic skeleton [Cu_2_(CA)(H_2_O)_2_, Cu-MOF-1] with polylactic acid (PLA) composite fiber membranes was prepared by Liu et al. [19]. Compared with commercially available copper nanoparticles (Cu-NPs), copper citrate and citric acid, Cu-MOF-1 exhibited better antimicrobial peculiarity, with inhibition rates of 99.3% and 97.9% against *S. aureus* and *Escherichia coli* (*E. coli*), respectively, when a dose of 250 μg·mL^−1^ was applied. The effect of the Cu-MOF-1/PLA fibrous membranes on *S. aureus* and *E. coli* also showed good bactericidal activity, with antibacterial rates of 99.8% and 99.3%, respectively [20,21].

In order to explore the antibacterial properties of a metal–organic framework, we selected a carboxylic acid ligand H_2_L, synthesized two new Cd(II) and Ni(II) compounds by adjusting the solvent ratio and changing the central metal atom, and discussed their coordination mode and structure. At the same time, PXRD, thermogravimetric, infrared and solid antibacterial properties of the synthesized compounds were tested, which further expanded the application prospect of the coordination polymer. In this paper, we discussed in detail the antibacterial properties of the two coordination polymers against *E. coli*, *S. aureus* and *C. albicans*. The experiments showed that the antibacterial properties of the two complexes were selective and showed good inhibitory properties against *S. aureus*. When the concentrations of coordination polymer 1 and 2 were 500 μg/mL and 400 μg/mL, respectively, the growth of *S. aureus* was almost completely inhibited within 24 h.

## 2. Results and Discussion

### 2.1. Structure Description of Compound ***1***

**Figure 1:**
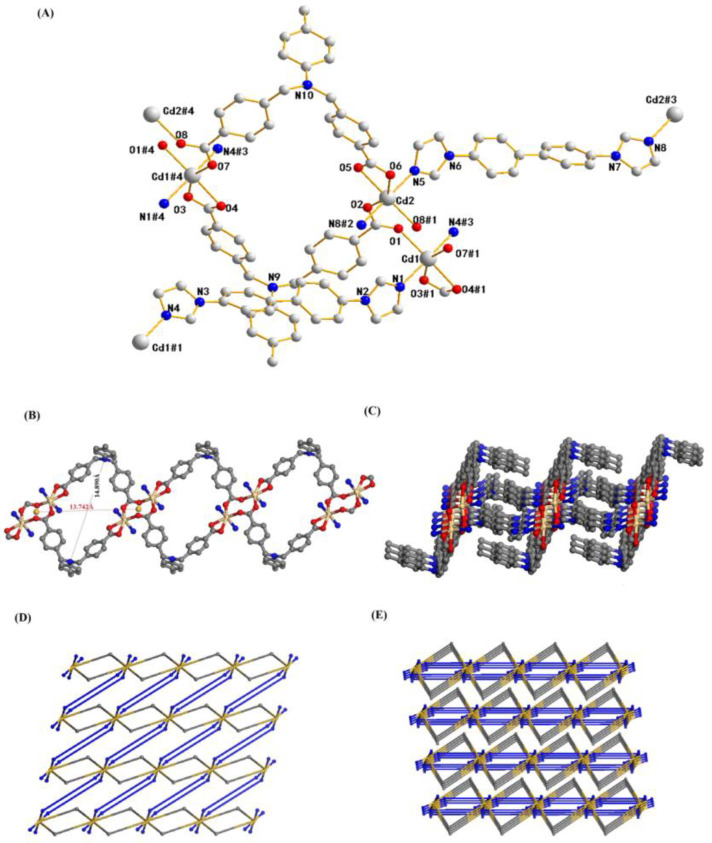
(**A**) Coordination environment of the Cd(II) ions in compound **1**. (**B**) 1D cyclic structure composed of carboxylic acid ligands. (**C**) 2D layered framework structure. (**D**) 2D layered topology. (**E**) 3D network structure with layer-by-layer stacking.

Single-crystal diffraction analysis indicates that compound **1** crystallizes in the Triclinic crystal system, *P1* space group. As shown in Figure 1A, the asymmetric motif in its configuration includes two crystallographically independent Cd(II) ions, two carboxylic acid H_2_L ligands, and two molecules of bibp ligands. In the asymmetric structural unit, both Cd(II) ions are in a hexacoordination mode, with Cd1 coordinating and bonding with O1, O3#1, O4#1 and O7#1 (from the two H_2_L ligands) and N1 and N4#3 (from the same nitrogen-containing ligand), and Cd_2_ coordinating and bonding with O2, O5, O6 and O8#1 (from the two H_2_L ligands) and N5 and N8#2 (from the same nitrogen-containing ligands), resulting in an irregular octahedral geometrical configuration, with the four oxygen atoms approximated to be considered co-planar and the nitrogen atoms in the axial position of the octahedron, where the Cd-O bonding distances are 2.222(10)–2.379(9) Å, the Cd-N bonding distances are 2.255(11)–2.371(9) Å, and the angles of N(1)-Cd(1)-N(4)# and N(5)-Cd(2)-N(8)#2 are 170.4° and 172.9°, respectively (Table S1, ESI†). In the asymmetric structural unit, the two carboxyl groups of the two-molecule carboxylic acid H_2_L ligand adopt different coordination modes, and they are bonded in a bidentate chelating and a monodentate cis–trans bridging sum, respectively. In this complex, the carboxylic acid H_2_L ligand forms a 1D ring structure with a hole of 13.742 Å × 14.890 Å with Cd(II) atoms (Figure 1B), and this 1D ring is then followed by two molecules of nitrogen-containing ligand bibp connected by Cd(II) to form a 2D layer (Figure 1C).

In order to represent its framework composition more simply, we regard the Cd(II) ion as a six-connected node, and the carboxylic acid ligand H_2_L and the nitrogen-containing ligand bibp as two-connected linkers; then, this 2D layer structure can be simplified into the topology shown in Figure 1D. Further software analysis shows that the same 2D layers eventually form a 3D network structure by π–π stacking (Figure 1E).

### 2.2. Structure Description of Compound ***2***

**Figure 2:**
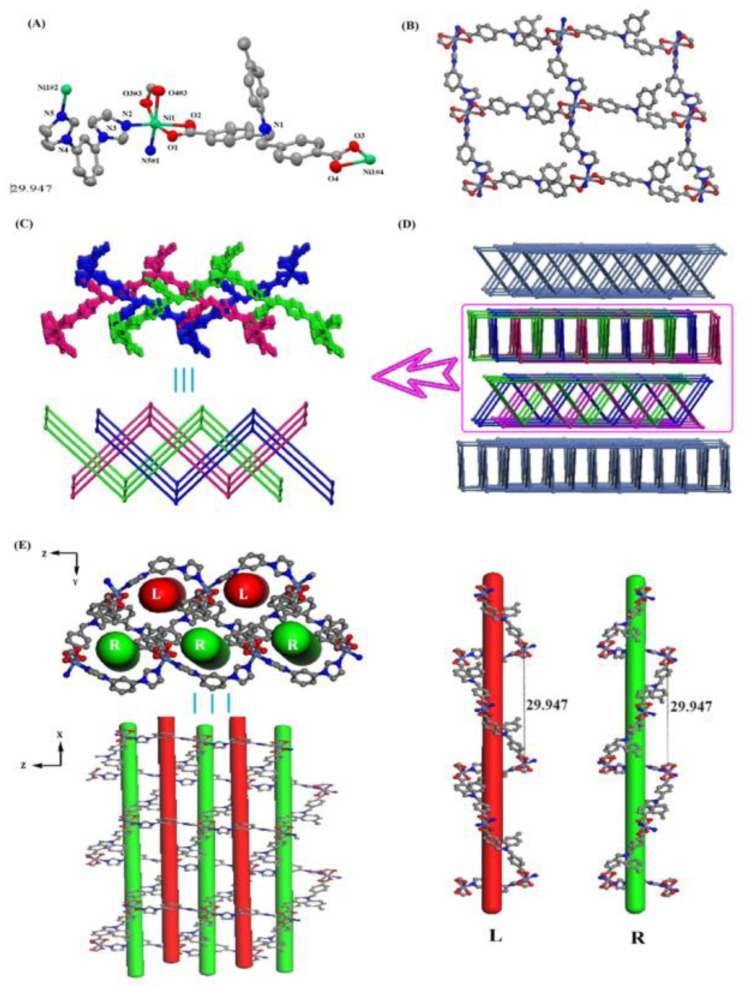
(**A**) Coordination environment of the Ni(II) ions in compound **2**. (**B**) 2D layered structure. (**C**) Triple-interpenetrated 2D layered structure and topology. (**D**) 3D network structure consisting of ABAB layer-by-layer stacking. (**E**) Helical chains within 2D layers.

Single-crystal diffraction analysis indicates that compound **2** crystallizes in the orthorhombic crystal system, *Fdd2* space group. As exhibited in Figure 2A, the asymmetric motif in its configuration contains a crystallographically independent Ni(II) ion, a molecule of carboxylic acid H_2_L ligand, and a molecule of bib ligand. In the asymmetric structural unit, the Ni(II) ion is coordinately bonded to four O atoms from two molecules of the carboxylic acid ligand (O1, O2, O3#3, O4#3) and two N atoms from two molecules of the nitrogen-containing ligand (N2, N5#1), respectively, constituting a deformed octahedral geometrical configuration, with the three oxygen atoms and the one nitrogen atom approximately coplanar and the rest of the ligand atoms in axial positions,. The Ni-O bond distance is 2.045(3)–2.175(4) Å, the Ni-N bond distance is 2.016(5)–2.059(4) Å, the angle of N(2)-Ni(1)-N(5)#1 is 94.81°, and the angle of N(2)-Ni(1)-O(1) is 156.26° (Table S1, ESI†). In the asymmetric structural unit, both carboxyl groups of the carboxylic acid H_2_L ligand adopt a bidentate chelate coordination mode to participate in bonding.

In this polymer, the carboxylic acid H_2_L ligand and the nitrogen-containing bib ligand are connected by Ni(II) ions to form two 1D cyclic chains, and the two 1D chains are then connected by unsaturated sites on the Ni(II) ions to form a 2D lattice structure (Figure 2B). Due to the large pores of the constituent lattice, the three identical 2D layers are interspersed with each other, forming a triple-interspersed 2D layer with a pattern of 2D plus 2D to 2D (2D + 2D → 2D) (Figure 2C), which is a relatively common pattern of interspersing.

We simplified the Ni(II) ion into a four-connected node, and the carboxylic acid ligand H_2_L and the nitrogen-containing ligand bib into two kinds of two-connected linkers; then, this 2D layer structure can be simplified into a topology as shown in Figure 2C (lower part). Careful analysis reveals that this triply interspersed 2D layer of the composition in turn forms a 3D network structure in an ABAB layer-by-layer stacking fashion (Figure 2D). In this compound, we further analyzed and found two helical chains in a separate 2D layer consisting of H_2_L ligand and bibp ligand with Ni(II) ions in the smallest repeating unit as [-Ni(L)_2_-Ni(bib)-] with a length of 29.947 Å, which were structurally diagrammed as shown in Figure 2E when viewed from the x-axial and y-axial directions.

### 2.3. PXRD and Thermogravimetric Analysis (TGA)

**Figure 3:**
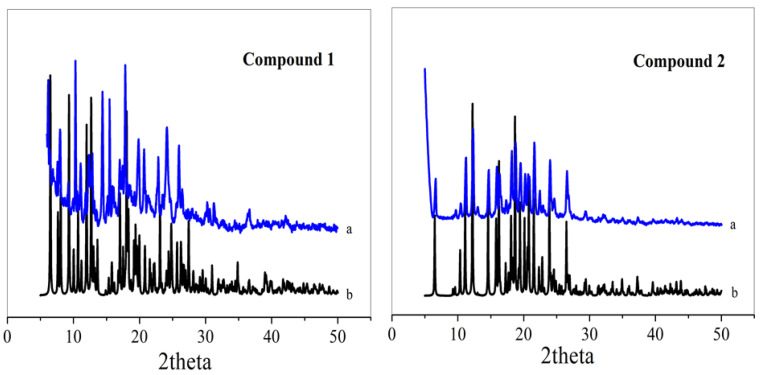
XRD patterns of compound **1** and **2** (a represents experimental XRD, b represents theoretical XRD).

To test the purity of the samples, the PXRD spectra of the samples were tested at room temperature. As can be seen in Figure 3, the locations of the diffraction peaks in the powder XRD spectra of samples **1** and **2** are essentially the same as those of the XRD spectra simulated by single-crystal structure data, which proves that the resulting target product is a pure phase.

**Figure 4:**
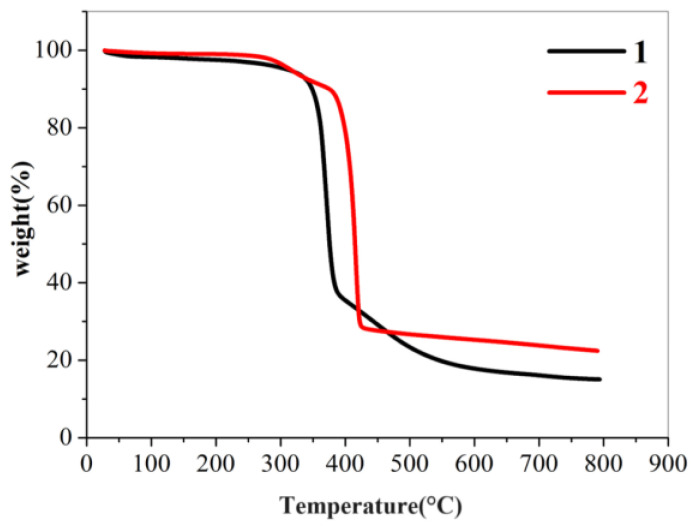
TGA patterns of compound **1** and **2**.

To test the thermal stability of compounds **1** and **2**, TGA experiments were carried out in a nitrogen atmosphere at a ramp rate of 10 °C/min from 20 to 800 °C. Moreover, **1** was stable up to 340 °C and **2** was stable up to 330 °C (Figure 4). The subsequent processes involved the thermal decomposition of the ligands and the collapse of the frameworks, respectively.

### 2.4. Culture of Bacteria and Fungi

In the experiment, the pre-configured PDB medium, LB medium and experimental vessels were autoclaved and transferred to an ultra-clean bench, after which the selected *Escherichia coli* (*E. coli*, BNCC 336902), *Staphylococcus aureus* (*S. aureus*, BNCC 330041), and *Candida albicans* (*C. albicans*, ATCC 10231) were inoculated into the culture solution and placed on the shaking table at 37 °C for overnight incubation. The culture was incubated overnight at 37 °C in a shaker. Finally, the inoculated bacteria were diluted 100-fold with culture medium.

### 2.5. Circle of Inhibition Test

In the circle of inhibition experiment, moderate amounts of *S. aureus* and *E. coli*, which use the culture medium diluted to 10^7^ CFU/mL, were uniformly applied to the LB medium, and *C. albicans* was applied to the PDB medium, so that it was completely absorbed. Immediately thereafter, a round paper sheet with a diameter of 6 mm was placed in an aqueous solution of either 1 mg/mL and 5 mg/mL of the compounds. Ultrasonic shaking was performed to make the complexes uniformly attach to the surface of the small paper sheet, and the sheet was then placed on the surface of the culture medium. Blank and carboxylic acid ligand control experiments were conducted using the same method. After that, the samples were put into a constant temperature incubator and incubated at 37 °C for 24 h, during which the inhibition was observed and the diameter of the inhibition circle was recorded. In order to ensure the rigor of the experiment, we repeated the bacteriostatic zone experiment three times.

**Figure 5:**
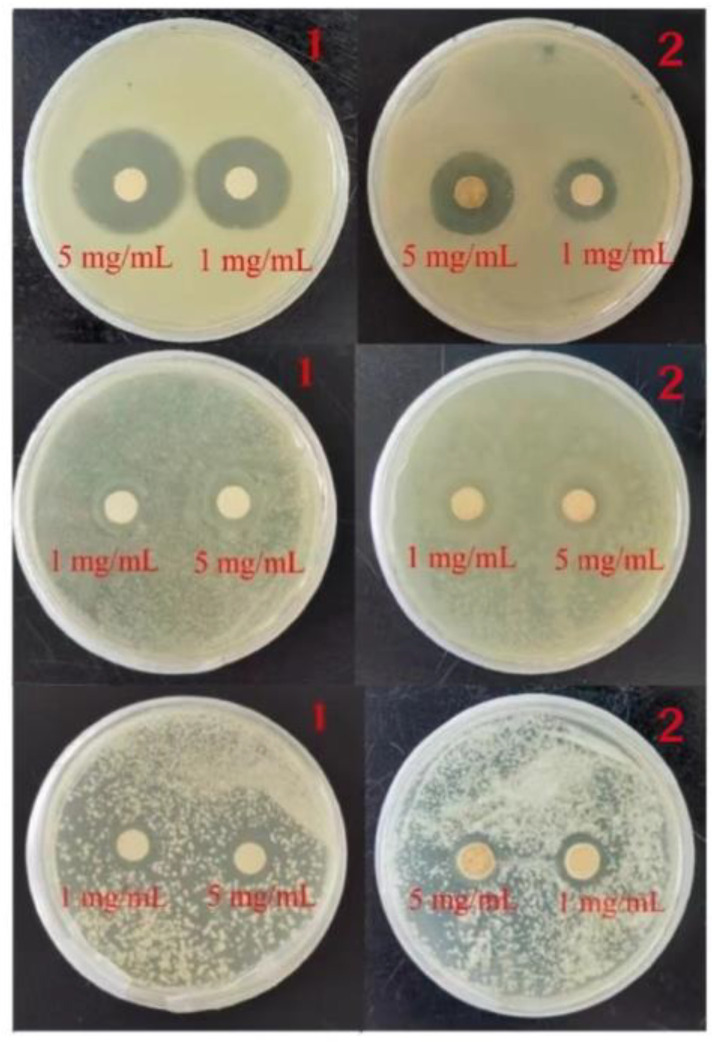
Inhibition zone of compound **1** and **2** against *S. aureus*, *E. coli* and *C. albicans* (**top** to **bottom**).

**Figure 6:**
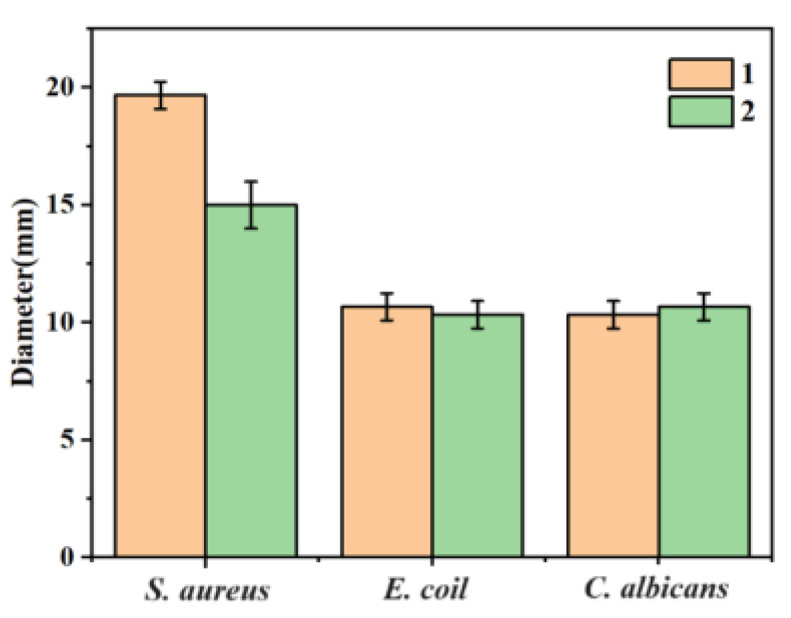
Histogram of the diameter of the circle of inhibition of compound **1** and **2** against *E. coli*, *S. aureus* and *C. albicans*.

Figure 5 shows that compounds **1** and **2** have different inhibitory effects on *E. coli, S. aureus* and *C. albicans*. A ratio of 5 mg/mL of compounds **1** and **2** showed a circle of inhibition diameter of 20 mm and 15 mm for *S. aureus*, and 11 mm and 9 mm for *E. coli*. For *C. albicans*, 5 mg/mL of compounds **1** and **2** showed a circle of inhibition of 10 mm and 11 mm. The average data of the three repeated experiments were summarized in the histogram of circle of inhibition diameters for *E. coli, S. aureus* and *C. albicans* plotted for each of the different compounds (Figure 6).

### 2.6. Growth Curve Experiment

**Figure 7:**
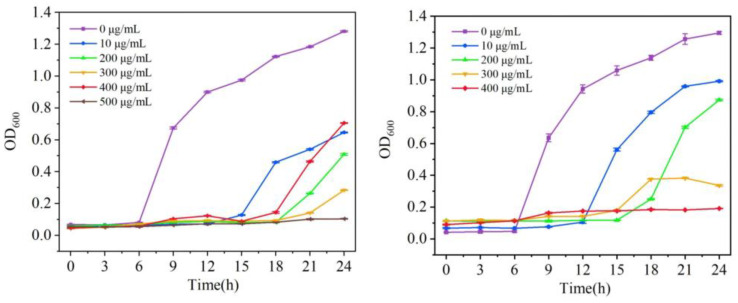
Growth curves of compound **1** and **2** against *S. aureus*.

The number concentration in the bacterial solution was measured with an enzyme marker at intervals. The survival of bacteria in the bacterial solution can be judged by the change in OD value. The growth curve of different concentrations of compounds **1** and **2** against *S. aureus* was plotted based on the average data of three repeated experiments (Figure 7).

The overnight bacterial solution was diluted into LB and PDB medium with different concentrations of compounds, and 200 μL of it was put into 96-well plates. An enzyme marker was used to determine the initial concentration of the bacterial solution. The bacterial solution was incubated in a shaker at 37 °C, and the absorbance was measured at 600 nm every 3 h with an enzyme labeler to determine the 24 h growth curve of the strain. In order to ensure the rigor of the experiment, we repeated the growth curve experiment three times.

**Figure 8:**
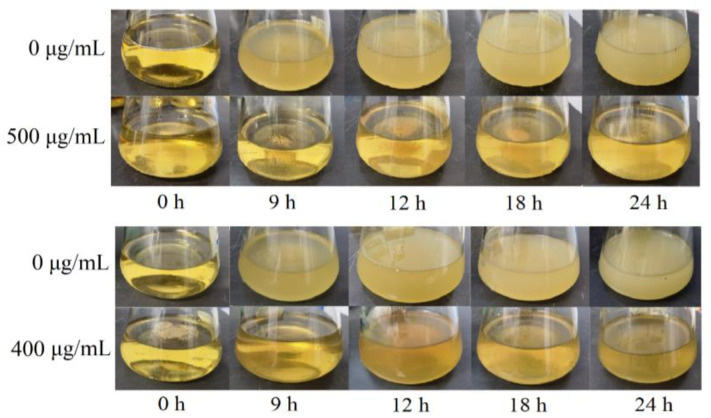
Changes in the bacteriostatic effect of compound **1** and **2** on *S. aureus* over 24 h.

The growth curves in Figure 7 show that the bacterial solution of the blank control group grew exponentially. For compounds **1** and **2**, the inhibitory effect was gradually enhanced with the gradient increase of the compound concentration, and when the concentration of the compounds reached 500 μg/mL and 400 μg/mL, respectively, the concentration of *S. aureus* liquid was no longer growing, and it was determined that it had been completely inhibited. According to the bacterial solution at different moments shown in Figure 8, with the change in time, the bacterial solution without the addition of the compound was gradually turbid; the bacterial solution with a high concentration of the compound formed a sharp contrast with the blank bacterial solution; the solution was more clarified, indicating that the concentration of the bacterial solution did not grow, which confirmed that compounds **1** and **2** had good antimicrobial properties against *S. aureus*.

**Figure 9:**
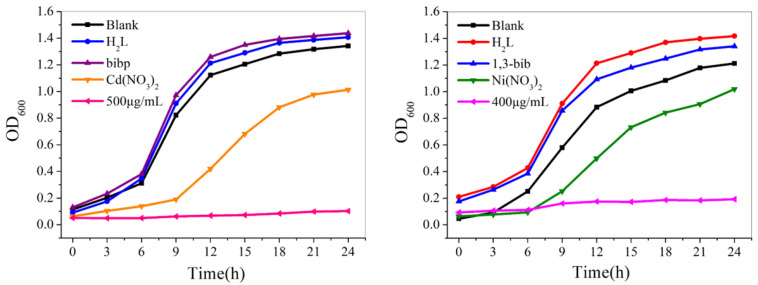
Growth curves of *S. aureus* by carboxylic acid ligands, nitrogenous ligands, metal salts, and compounds.

At the same time, comparative experiments were carried out. For compound **1**, the growth curves of *S. aureus* were tested in blank, H_2_L, bibp, Cd(NO_3_)_2_, and compound against *S. aureus* when the concentration was 500 μg/mL. For compound **2**, the growth curves of *S. aureus* were similarly tested in blank, H_2_L, bib, Ni(NO_3_)_2_ and compound when the concentration was 400 μg/mL. As shown in Figure 9, it was observed that the concentration of the bacterial solution containing carboxylic acid ligands, nitrogen-containing ligands, and metal salts increased exponentially with increasing time, while the concentration of the bacterial solution containing the compounds remained unchanged, indicating that it was the compounds that inhibited the growth of the bacterial solution.

## 3. Experimental Section

### 3.1. Materials and Methods

**Figure 10:**
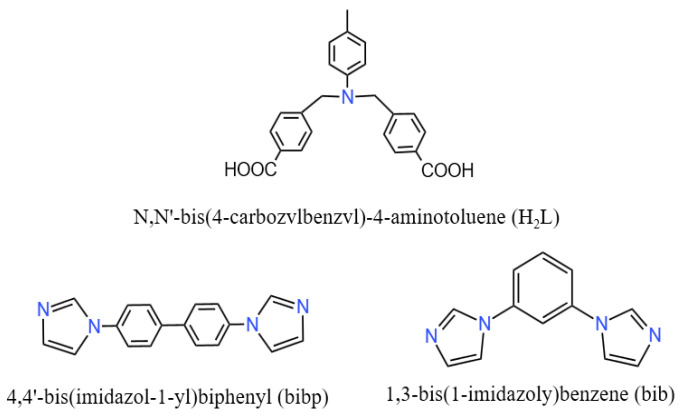
Molecular structure of ligands.

The ligands H_2_L, bibp, and bib were purchased from Jinan Henghua SCI. & TEC. Co., Ltd. (Figure 10) (in Jinan, Shandong Province, China). Other raw materials were of analytical level and used without further purification. Powder X-ray diffraction (PXRD) data were collected on a D2 Phaser A26-X1 XRD diffraction meter. IR spectra (4000-400 CM^−1^) were obtained from KBr particles with an FT-IR Nexus spectrometer. Element analysis was performed on a Perkin-Elmer 240 C analyzer. The thermostat analysis (TGA) curve was determined at a heating rate of 10 °C/min on a Perkin-Elmer TG-7 thermal analyzer. A Spectra Max Plus 384 microplate reader was used to determine OD600 of the bacterial liquid.

### 3.2. Synthesis of [Cd_2_(L)_2_(bibp)_2_]_n_ (***1***)

A mixture of Cd(NO_3_)_2_·4H_2_O (0.0308 g, 0.1 mmol), H_2_L (0.0375 g, 0.1 mmol) and bibp (0.0286 g, 0.1 mmol ) were dissolved in the DMF/H_2_O solvent (7:1, 8 mL). After mixing evenly, the solution was placed in a Parr Teflon-lined stainless-steel vessel (20 mL) and heated at 140 °C for 72 h under autogenous pressure. Colorless crystals were acquired. The reaction yield was ca. 49% based on bibp. Anal. calcd for C_82_H_66_N_10_O_8_Cd_2_ (%): C, 61.58; H, 4.23; N, 9.11. Found: C, 63.78; H, 4.31; N, 9.07. FT-IR (4000-400 cm^−1^): 3416(s), 1597(s), 1519(s), 1385(m), 1350(m), 1303(w), 1123(s), 1058(m), 962(s), 822(s), 794(s), 771(s), 655(s).

### 3.3. Synthesis of [Ni(L)(bib)]_n_ (***2***)

A mixture of Ni(NO_3_)_2_·6H_2_O (0.0291 g, 0.1 mmol), H_2_L (0.0375 g, 0.1 mmol) and bib (0.0211 g, 0.1 mmol) were dissolved in the H_2_O solvent (10 mL). After mixing evenly, it was placed in a Parr Teflon-lined stainless-steel vessel (20 mL) and heated at 140 °C for 72 h under autogenous pressure. Green crystals were acquired. The reaction yield was ca. 51% based on bibp. Anal. calcd for C_35_H_29_N_5_O_4_Ni (%): C, 64.36; H, 4.37; N, 10.56. Found: C, 65.45; H, 4.55; N, 10.90. FT-IR (4000-400 cm^−1^): 3421(s),1594(m), 1522(m), 1385(m), 1350(s), 1017(s), 941(s), 771(s), 707(s), 619(s).

### 3.4. X-ray Crystallography

The data on the crystal structure of compounds **1** and **2** were collected at room temperature using a Bruker SMART APEX II CCD X-ray single-crystal diffractometer from Bruker, Germany, with a radiation source of Mo *Kα* rays (*λ* = 0.071073 nm). The diffraction data were reduced and refined using the SAINT program, the crystal structure was solved by direct method from the Olex program, the hydrogen atoms were determined by theoretical hydrogenation, and all non-hydrogen atom coordinates and their anisotropic thermal parameters were found using the full-matrix least-squares method. The resulting crystal information for compound **1** and **2** is shown in Table 1.

## 4. Conclusions

In this article, two new coordination polymers were synthesized under solvent thermal and hydrothermal conditions, and their antibacterial properties were studied. Antibacterial performance experiments have shown that compounds **1** and **2** have certain selectivity in their antibacterial properties and have good antibacterial properties against *S. aureus*, but their antibacterial effects on other microorganisms are not as expected. As the concentration of each compound increases, the inhibitory effect gradually strengthens, and when the concentration reaches 500 μg/mL and 400 μg/mL for compound **1** and **2**, respectively, the concentration of the *S. aureus* solution no longer increases and is completely inhibited. Due to the excellent stability of the coordinating polymer itself, it exhibits excellent antibacterial effects within 24 h. The active site of the compound can destroy the cell membrane to a certain extent after binding with *S. aureus*, thus accelerating the death of the bacteria.

## Figures and Tables

**Table 1:** Crystal and structure refinement data for compound **1** and **2**.

Compound	1	2
CCDC No.	2338641	2338643
Molecular Formula	C_82_H_66_N_10_O_8_Cd_2_	C_35_H_29_N_5_O_4_Ni
*F*_w_	1544.27	642.32
Crystal system	Triclinic	Orthorhombic
Space group	P1	Fdd2
*a*/Å	10.9792(7)	19.6165(14)
*b*/Å	11.6582(8)	54.500(4)
*c*/Å	13.7423(9)	11.1375(8)
*α*/(°)	81.8140(10)	90
*β*/(°)	86.0920(10)	90
*γ*/(°)	88.6630(10)	90
V/Å3	1736.8(2)	11907.2(15)
*Z*	1	16
*D*_c_/(g⋅cm^−3^)	1.897	1.615
*F* (000)	920	5760
GOF on *F*^2^	1.038	1.037
*R*_1_*/wR*_2_ [*I* > 2*σ*(*I*)]	0.0347, 0.0756	0.0574, 0.1009
*R*_1_*/wR*_2_ (all data)	0.0496, 0.0819	0.1251, 0.1319

## Data Availability

Data are contained within the article and Supplementary Materials.

## Supplementary Materials

The following supporting information can be downloaded at: https://www.mdpi.com/article/10.3390/molecules29091990/s1.
