# Supplementary material for: Synthesis, Structure and Antibacterial Activity of Two Novel Coordination Polymers Based on N,N′-bis(4-carbozvlbenzvl)-4-aminotoluene and Heterocyclic Ligand against S. aureus
Source: Molecules. 2024 Apr 26;29(9):1990. doi: 10.3390/molecules29091990 (PMC11085063; doi:10.3390/molecules29091990)
Supplement: Supplementary file 1 [file molecules-29-01990-s001.zip › molecules-2950928-supplementary.pdf]

Synthesis, Structure and Antibacterial Activity of Two Novel Coordination Polymers Based on *N,N′*-bis(4-carbozvlbenzvl)-4-aminotoluene and Heterocyclic Ligand against *S. aureus*

Ziyun Wang, Lun Zhao *, Hongwei Jing, Guanying Song and Jiayu Li

College of Chemistry, Changchun Normal University, Changchun 130032, China; qx202200053@stu.ccsfu.edu.cn (Z.W.); qx202100047@stu.ccsfu.edu.cn (H.J.); qx202100049@stu.ccsfu.edu.cn (G.S.); lijiayu@stu.ccsfu.edu.cn (J.L.)

* Correspondence: zhaolun7511@126.com; Tel.: +86-431-86168903

Table S1. Selected Bond Lengths (Å) and Angles (deg) for **1** and **2**

Compound **1**			
O(3)-Cd(1)#4	2.379(9)	Cd(2)-O(5)	2.367(9)
O(4)-Cd(1)#4	2.379(8)	Cd(2)-N(8)#2	2.371(9)
O(7)-Cd(1)#4	2.222(10)	Cd(2)-O(6)	2.394(7)
O(8)-Cd(2)#4	2.246(9)	Cd(1)-O(7)#1	2.222(10)
N(4)-Cd(1)#2	2.332(10)	Cd(1)-O(1)	2.301(9)
N(8)-Cd(2)#3	2.371(9)	Cd(1)-N(1)	2.330(10)
Cd(2)-O(2)	2.220(10)	Cd(1)-N(4)#3	2.332(10)
Cd(2)-O(8)#1	2.246(9)	Cd(1)-O(3)#1	2.379(9)
Cd(2)-N(5)	2.255(11)	Cd(1)-O(4)#1	2.379(8)
O(2)-Cd(2)-O(8)#1	102.8(3)	O(7)#1-Cd(1)-O(1)	102.9(3)
O(2)-Cd(2)-N(5)	96.6(4)	O(7)#1-Cd(1)-N(1)	99.0(4)
O(8)#1-Cd(2)-N(5)	95.5(4)	O(1)-Cd(1)-N(1)	96.0(3)
O(2)-Cd(2)-O(5)	96.0(3)	O(7)#1-Cd(1)-N(4)#3	90.6(4)
O(8)#1-Cd(2)-O(5)	158.6(3)	O(1)-Cd(1)-N(4)#3	82.1(3)
N(5)-Cd(2)-O(5)	92.4(4)	N(1)-Cd(1)-N(4)#3	170.4(4)
O(2)-Cd(2)-N(8)#2	90.4(3)	O(7)#1-Cd(1)-O(3)#1	154.3(3)
O(8)#1-Cd(2)-N(8)#2	83.3(3)	O(1)-Cd(1)-O(3)#1	101.8(3)
N(5)-Cd(2)-N(8)#2	172.9(4)	N(1)-Cd(1)-O(3)#1	85.4(3)
O(5)-Cd(2)-N(8)#2	86.5(3)	N(4)#3-Cd(1)-O(3)#1	85.8(3)
O(2)-Cd(2)-O(6)	151.0(3)	O(7)#1-Cd(1)-O(4)#1	98.1(3)
O(8)#1-Cd(2)-O(6)	105.5(3)	O(1)-Cd(1)-O(4)#1	156.4(3)
N(5)-Cd(2)-O(6)	86.8(4)	N(1)-Cd(1)-O(4)#1	91.2(3)
O(5)-Cd(2)-O(6)	55.1(3)	N(4)#3-Cd(1)-O(4)#1	87.0(3)
N(8)#2-Cd(2)-O(6)	86.8(3)	O(3)#1-Cd(1)-O(4)#1	56.3(3)
Symmetry codes:#1 x,y,z+1; #2 x-1,y,z-1; #3 x+1,y,z+1; #4 x,y,z-1			
Compound **2**			
Ni(1)-N(2)	2.016(5)	Ni(1)-O(1)	2.167(4)
Ni(1)-O(3)	2.045(3)	Ni(1)-O(4)	2.175(4)
Ni(1)-N(5)#1	2.059(4)	N(5)-Ni(1)#2	2.059(4)

Ni(1)-O(2)	2.066(3)		
O(3)-Ni(1)-O(4)	62.00(13)	N(5)#1-Ni(1)-O(2)	99.63(15)
N(5)#1-Ni(1)-O(4)	158.85(15)	N(2)-Ni(1)-O(1)	156.26(16)
O(2)-Ni(1)-O(4)	99.82(14)	O(3)-Ni(1)-O(1)	104.01(14)
O(1)-Ni(1)-O(4)	89.71(15)	N(5)#1-Ni(1)-O(1)	92.02(15)
N(2)-Ni(1)-O(3)	97.66(16)	O(2)-Ni(1)-O(1)	62.29(14)
N(2)-Ni(1)-N(5)#1	94.81(18)	N(2)-Ni(1)-O(4)	92.00(17)
O(3)-Ni(1)-N(5)#1	97.20(15)	O(3)-Ni(1)-O(2)	158.53(13)
N(2)-Ni(1)-O(2)	94.12(16)		
Symmetry codes: #1 x,y,z+1； #2 x,y,z-1；#3 x+3/4,-y+1/4,z-1/4；#4 x-3/4,-y+1/4,z+1/4			

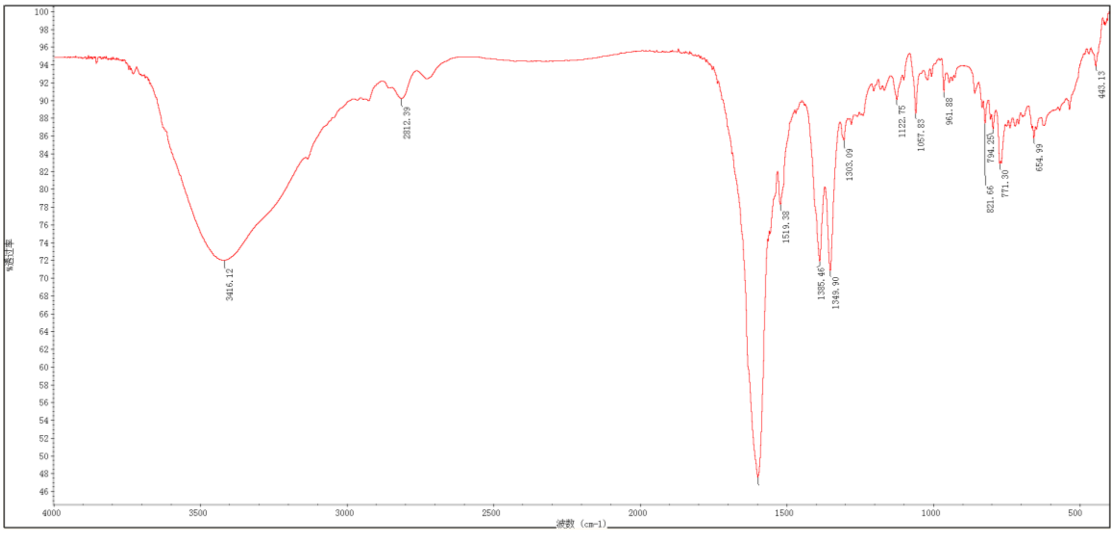

Figure S1. IR spectra of Compound 1.

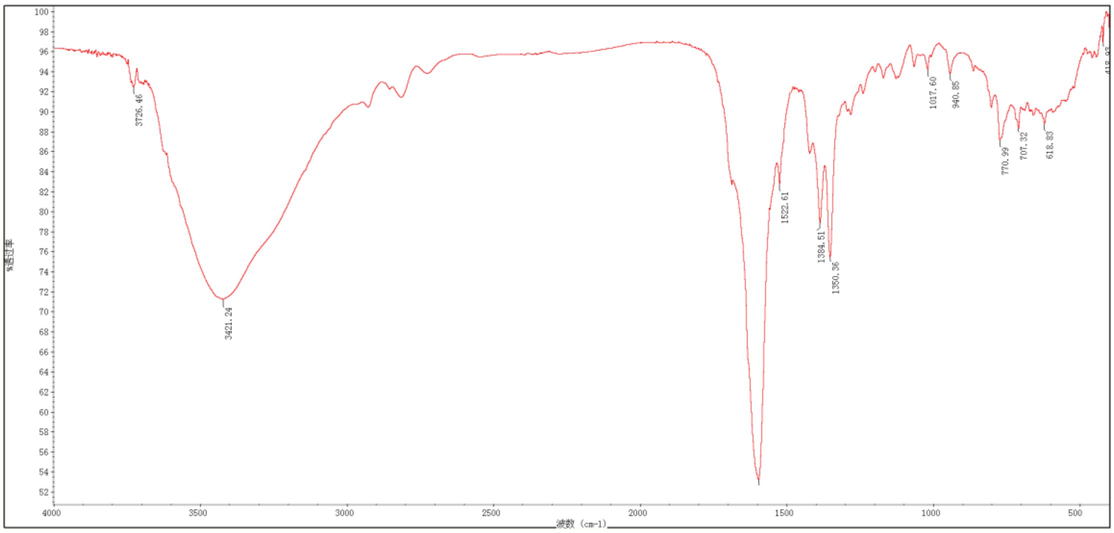

Figure S2. IR spectra of Compound 2.
